# Phylogenetics reveals the crustacean order Amphionidacea to be larval shrimps (Decapoda: Caridea)

**DOI:** 10.1038/srep17464

**Published:** 2015-12-08

**Authors:** Sammy De Grave, Tin-Yam Chan, Ka Hou Chu, Chien-Hui Yang, José M. Landeira

**Affiliations:** 1 Oxford University Museum of Natural History, Oxford, United Kingdom; 2 Institute of Marine Biology, National Taiwan Ocean University, Keelung 20224, Taiwan; 3 Center of Excellence for the Oceans, National Taiwan Ocean University, Keelung 20224, Taiwan; 4 Simon F.S. Li Marine Science Laboratory, School of Life Sciences, The Chinese University of Hong Kong, Hong Kong, China; 5 Shenzhen Research Institute, The Chinese University Hong Kong, Shenzhen, China; 6 Graduate School of Fisheries Sciences, Hokkaido University, 3-1 Minato, Hakodate, Hokkaido 041-8611, Japan

## Abstract

We present evidence that the single representative of the crustacean order Amphionidacea is a decapod shrimp and not a distinct order. After reviewing available morphological evidence, it is concluded that *Amphionides* is a larval form, but with an as yet unknown parentage. Although the most likely adult form is in the family Pandalidae, the limited molecular data available cannot fully resolve its affinity. We therefore propose to treat *Amphionides reynaudii* as *incertae sedis* within Caridea, rather than a separate family. In view of the large scale, tropical and subtropical distribution of the taxon, the possibility is discussed that *Amphionides* is more likely to be a composite taxon at generic level, rather than larvae of a single shrimp species.

In the traditional classification scheme of crustaceans^1^, the crustacean superorder Eucarida comprises three orders, Euphausiacea, Amphionidacea and Decapoda, although the relationship of euphausiids to decapods remains unclear^2^. *Amphionides*, the sole genus in the order Amphionidacea is typically planktonic, and found in all subtropical-tropical oceans^3^, with presumed larval specimens usually collected at depths less than 100 m, whilst metamorphosed specimens are mostly found below 700 m. Historically, four larval nominal taxa were recognised in the genus, as well as an additional metamorphosed nominal taxon. However, these have all been considered to be synonyms of each other since 1969^4^, and currently only one species is recognised in the order. The majority of specimens were collected during major historical oceanographic expeditions, such as the Dana expedition^4^ and the International Indian Ocean Expedition^5^, with very few specimens recorded since 1973^3^. Most specimens are rather damaged (Fig. 1), with no supposed intact adult specimen ever collected.

The order Amphionidacea was erected in 1973^5^ and is currently assumed to consist of only a single species, *Amphionides reynaudii* (H. Milne Edwards, 1833), but a separate ordinal status has been controversial for many decades. Although large-scale compilations and summaries of crustacean systematics have followed Williamson’s concept^1^,^3^,^6^,^7^, Richter & Scholtz^2^ mentioned briefly that the order should be included in the Decapoda, a view shared by many of the earlier crustacean workers. Koeppel in 1902 saw affinities with larvae of the Sergestidae^8^, whilst even earlier in 1888 Spence Bate^9^ considered it closely related to the larval genus *Eretmocaris* (now considered to be the larvae of the caridean genus *Lysmata*). Gurney, who was an expert in the morphology of larval Decapoda, considered the genus to be clearly based within Caridea^10^ and in 1942 placed the genus in its own family, Amphionidae (recte Amphionididae)^11^. Based on an exhaustive study of the Dana expedition material Heegaard^4^, whilst maintaining the family, placed it as the sole member of the sub-tribe Amphionidea within the tribe Caridea, although indicating that it may well constitute a tribe on its own.

Despite the interesting morphology of the group, the position of *Amphionides* has only been scrutinised three times within an analytical framework; all have been based on morphology as to date no material suitable for genetics has come to light. In an early cladistic analysis of Eucarida, Amphionidacea was resolved as the sister-group to Decapoda^12^, a relationship that was resolved also in an expanded analysis of Malacostraca^2^. However, the latter study in a summary of evolutionary relationships suggested that ‘the Decapoda should also comprise the Amphionidacea’. In a more recent, exhaustive analysis of recent and fossil Arthropoda, the same sister-relationship was recovered^13^.

Recently freshly-collected material of *A. reynaudii* from the Canary Islands (Atlantic Ocean) has become available, and we are now able to elucidate the phylogenetic position of the taxon on the basis of four molecular markers. DNA analysis has in recent years proved to be a powerful tool for linking enigmatic larval or planktonic forms to adult taxa, especially in decapod species with unknown larval taxa, an area where morphology often fails to correctly link both taxa. For example, the enigmatic deep-sea taxon, *Galatheacaris abyssalis* (previously placed in its own family and superfamily within Caridea^14^) represents the decapodid stage of *Eugonatonotus chacei*^15^. In a similar way, phylogenetics demonstrated that the armoured, pelagic taxon, *Cerataspis monstrosa* is a larval form of the wide-spread dendrobranchiate shrimp, *Plesiopenaeus armatus*^16^.

## Results

The final four- and two-marker datasets comprise 59 species each with 3884 bp and 205 species each with 2234 bp, respectively (Supplementary Figure S1 and Supplementary Table S2). A total of 95 species and 190 gene sequences (excluding *A. reynaudii*) are added to an existing two-marker dataset^17^. The optimal models for 16 S and 18 S alignments of the two-marker dataset assessed are all with gamma-distributed (G) and invariant sites (I). Tree topologies from ML and BI analyses are generally similar and the BI phylogram is presented to show the phylogenetic relationship of *A. reynaudii* in both the four- and two-marker results (Figs. 2 and 3).

Using Euphausiacea and Stomatopoda as non-decapod outgroups and including all the suborders and infraorders of decapods, as well as 19 species of carideans from nine families, the four-gene phylogenetic tree (Fig. 2) reveals that *Amphionides* is nested inside the carideans and sister to *Pandalus montagui* of the family Pandalidae (Pp = 1.0, MLb = 99), The nodal support for the major groups of decapod crustaceans is generally strong and similar to a previous analysis^18^. The result of the AU test rejects the hypothesis that *A. reynaudii* does not belong to Caridea (*P* = 0.027).

The two-marker analysis including 190 species of carideans in 29 families (out of 35 currently recognised) has the most comprehensive species level taxon coverage for carideans to date. This phylogenetic tree (Fig. 3, Supplementary Table S2) demonstrates that *A. reynaudii* is nested within Pandalidae, but with moderate support from ML (Pp = 1.0, MLb = 57). Although *A. reynaudii* is sister to the genus *Heterocarpus* (with three species in the present analysis), the support is not very strong (Pp = 1.0, MLb ≤ 50). Result of the AU test shows that the hypothesis that *A. reynaudii* does not belong to Pandalidae cannot be rejected (*P* = 0.145). The relationships among the various families of Caridea in our current analysis are similar to previous studies^17^,^19^ and demonstrate para- and polyphyly of certain families (e.g. Pasiphaeidae, Palaemonidae).

## Discussion

Our results demonstrate strongly that *Amphionides reynaudii* is a decapod (Fig. 2), and more specifically a caridean shrimp, and find no support for separate order status despite being considered a sister group to Decapoda on the basis of morphology^2^,^12^. In line with this result, we thus formally invalidate the order Amphionidacea Williamson, 1973. Accurately placing the taxon within Caridea is more problematic. Although the alignment suggests a close affinity to Pandalidae, the result of the AU test (*p* = 0.145) does not confirm the taxon as unambiguously within Pandalidae, although they are likely to be closely related. A potential close relationship between *Amphionides* and Pandalidae has already been suggested, primarily based on the morphology of younger larvae^4^.

The recovered placement of *Amphionides* as a caridean shrimp raises several questions. Only three adult males have ever been found^5^, although these are two specimens originally deemed female^10^,^20^ and a single early postlarval specimen (named postlarva I)^4^. All three were re-assigned as males^5^ based on the perceived advanced development of the testis and vasa differentia compared to earlier zoeal stages. Williamson^5^ further remarked that if they would not be sexually mature they then would indeed be decapodids (i.e. a late larval stage). However, sexually mature males in Caridea are characterised by the presence of an appendix masculina on the second pleopod^21^, with only two known exceptions, the genera *Synalpheus* (Alpheidae) and *Janicella* (Oplophoridae). According to Williamson^5^, sexual differentiation in *Amphionides* starts in zoeal stage IX, and two distinct morphotypes are present after this stage, based on the thickness of the antennular segment and flagellar length, with no distinction made on the basis of the second pleopod. In none of the specimens so far illustrated in the literature^4^,^5^,^10^,^20^,^22^ is an appendix masculina visible, nor is it discussed in the text. It further appears that no author has ever seen a fully intact adult female, with the well-known illustration in Williamson^5^ being a composite of 43 damaged specimens, considered to be female. Kutschera *et al.*^22^, on the basis of the developmental stages in Williamson^5^, did assume that one specimen in their study was male and one female, but made no comment on sexual maturity. It thus appears that the vast majority of reported specimens are larvae, with the purported sexually mature specimens based on somewhat dubiously interpreted internal characters. Gurney^10^ suggested that the observed testis and ovaries in previous studies were instead the posterior diverticula of the stomach. The lack of an appendix masculina in purported adult males, a near universal characteristic of Caridea, provides ample credence that no real adults have ever been found of *Amphionides*. Although an outside possibility remains that *Amphionides* is a neotenic form, on balance the available evidence overwhelmingly suggests that *Amphionides* is a larval form, not yet linked to a known adult form. Exotic larval morphologies abound in the Decapoda, for instance phyllosoma larvae, and a plethora of nominal taxa of larval forms not yet linked to adult taxa is known within Caridea^23^. In recent years, some of these forms have been linked to adult genera, either on the basis of direct rearing or molecular evidence. For instance, *Eretmocaris* was shown to be the zoeal stage of *Lysmata* based on laboratory rearing^24^, whilst COI data conclusively showed *Galatheacaris* to be the decapodid stage of *Eugonatonotus*^15^ and *Icotopus* to be the late larvae of *Plesionika*^25^.

On the hypothesis that *Amphionides* is a larval stage, a question is opened about the conspecificity of all specimens noted to date. At present, *A. reynaudii* is thought to occur in the Atlantic, Pacific and Indian Oceans^4^,^5^, although considered to be much more abundant in the Atlantic Ocean^4^. Nevertheless, early larval stages of *Amphionides* are rare among the material from both plankton and micronekton surveys around the Canary Islands and Cape Verde^26^,^27^, and no specimens were encountered in an analysis of the crustacean larval community based on extensive plankton samples collected in oceanic and coastal waters off the Canary Islands-African transition zone^28^.

Few caridean species exhibit such a broad distribution as *Amphionides*, although examples do exist in the Oplophoridae (e.g. *Acanthephyra eximia*), Pasiphaeidae (e.g. *Eupasiphaea gilesii, Glyphus marsupialis*), Palaemonidae (e.g. *Brachycarpus biunguiculatus, Gnathophyllum americanum*) and Pandalidae (e.g. *Chlorotocus crassicornis, Plesionika williamsi, Stylopandalus richardi*). At a generic level such a wide distribution is much more common, especially in deep water and/or pelagic taxa, in families like Pasiphaeidae, Acanthephyridae, Oplophoridae and Pandalidae. There is thus a possibility that the nominal taxa (originally described from different geographic regions) and currently considered to be synonyms of *A. reynaudii*, are perhaps larval stages of congeneric species, rather than conspecific. For instance, *Amphion reynaudii* H. Milne Edwards, 1832 was described from “les mers d’Asie”, whilst *Amphion provocatoris* Spence Bate, 1888 has “south of the Azores” as its type locality, approximately the area from which the present specimens were collected.

The possibility remains, however, that *Amphionides reynaudii* as presently understood is the larval phase of a single species. Records of the depth distribution of the taxon are somewhat conflicting, but in general it is known that earlier larval stages are present from the surface down to 30–100 m, with later stages primarily known from 700–1700 m^4^,^5^. However, the present records also demonstrate that later stages can occur in shallower water. A bathymetric range down to 2000–5000 m has been postulated^4^ and it has been suggested that the taxon undergoes diel vertical migration (DVM) towards the surface^4^. Decapod crustaceans are indeed a dominant component of the mesopelagic community occurring at those depths and undergoing DVM, with Oplophoridae, Pasiphaeidae and Pandalidae being prominent^29^.

Our molecular data point strongly towards *Amphionides* being allied with if not part of Pandalidae, to the exclusion of many families analysed (Fig. 3). Six currently recognised caridean families were not included in the present analysis, namely Bresiliidae, Bythocarididae, Campylonotidae, Merguiidae, Physetocarididae and Pseudochelidae. All of these are unlikely to be allied to *Amphionides*, in view of their restricted ecology (e.g. *Merguia*, the sole genus in Merguiidae is a tropical mangrove inhabitant), biogeography (e.g. *Campylonotus*, the sole genus in Campylonotidae is sub-Antarctic) or indeed have known larval stages (e.g. *Physetocaris*). Our analysis does recover Crangonidae as a sister group to Pandalidae, with morphological similarities between early larval stages of that family and *Amphionides* already noted^22^. On the whole, however, later stages of crangonid larvae are rather conservative across the family and show no similarity to *Amphionides*. Several molecular analyses place Pandalidae in a larger clade^17^,^19^, somewhat similar in composition to the one recovered herein, and consistently featuring Hippolytidae *sensu lato*, now split into several families^30^. Although larval morphologies for most families of carideans appear rather conservative, hippolytoids are an exception and show a wide diversity of larval forms, even though molecular evidence is not supportive of a close link between hippolytoids and *Amphionides*.

In relation to the hypothesis that *Amphionides* is indeed a larval pseudo-taxon, and not a neotenic one (in the absence of secondary sexual characteristics), the question as to which known caridean taxon is the adult form remains open, largely caused by the limited molecular data in Caridea as a whole, and particularly within the family Pandalidae, to which *Amphionides* is allied. The current analysis for example, only has representatives of three pandalid genera included, out of a total of 23 genera^23^. Within the area from which the present specimen was taken (the Canary Islands), several genera and species of Pandalidae occur^31^, be it benthic or pelagic, shallow or deeper water. For many of these genera, the larvae are tolerably well known^28^ and show no morphological similarity to *Amphionides*. However, two genera, *Bitias* and *Pantomus*, have unknown larval morphologies and potentially could be candidates to be the adult form of *Amphionides* although neither taxon exhibits the global distribution of *Amphionides* (at species or generic level) adding further to the puzzle.

On the basis of the above line of argumentation and in view of the inconclusive molecular data, we therefore elect to treat *Amphionides reynaudii* as *incertae sedis* within Caridea, pending further resolution. The genus has previously been placed in its own family within the Caridea, namely Amphionididae^11^, but this family could alternatively be added to the list of valid families in Caridea, which would effectively then make *Amphionides* a neotenic, planktonic form. However, making such a profound change to higher level systematics in Caridea appears premature, given the general unsettled relationships of caridean families and status as *incertae sedis* is considered to be a more elegant solution given the currently available data.

## Methods

### Specimens

Six specimens of *Amphionides reynaudii* were collected north of Gran Canaria, NE Atlantic (28°31′N, 15°22’W) on the 1^st^ April 2011. All six specimens were collected in the same trawl (Matsuda Oozeki Hu net, MOHT, 5 m^2^ net mouth, 4 mm mesh size). The trawl was taken one hour after the nocturnal ascent of the acoustic scattering layer from 150 m depth to the surface, with a towing speed of 3–4 knots^29^. All six specimens are larvae. Using the latest staging scheme^22^, two specimens (length of cephalothoracic shield (CtsL) at least 10.5 mm, anterior carapace detached; CtsL 12.5 mm, abdominal somite IV and telson missing) belong to stage 9 (thoracopod VIII present and all pleopods present), three specimens (CtsL 10.1, 11.2, 13.6 mm) belong to stage 8 (thoracopod VII present but thoracopod VIII absent, all pleopods present), and one specimen (CtsL 7.0 mm, without abdomen) belongs to stage 4 (thoracopod V moderately long). Specimens are deposited in the collection of the National Taiwan Ocean University (NTOU M01871, M01872).

**Figure 1:**
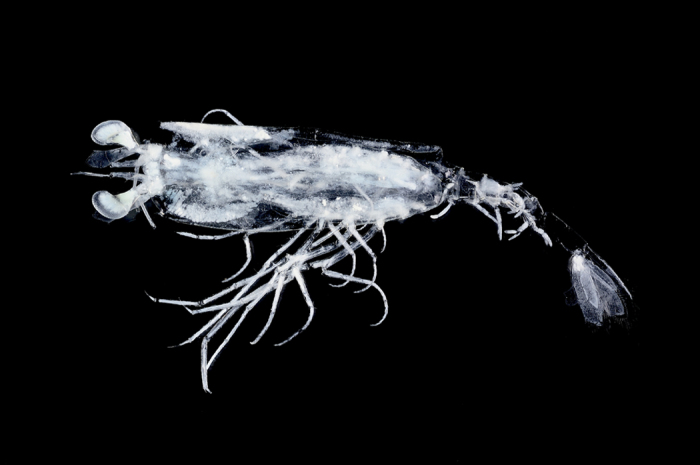
*Amphionides reynaudii* (H. Milne Edwards, 1833). Specimen sequenced herein, collected north of Gran Canaria, stage 8 larva (NTOU M10872).

**Figure 2:**
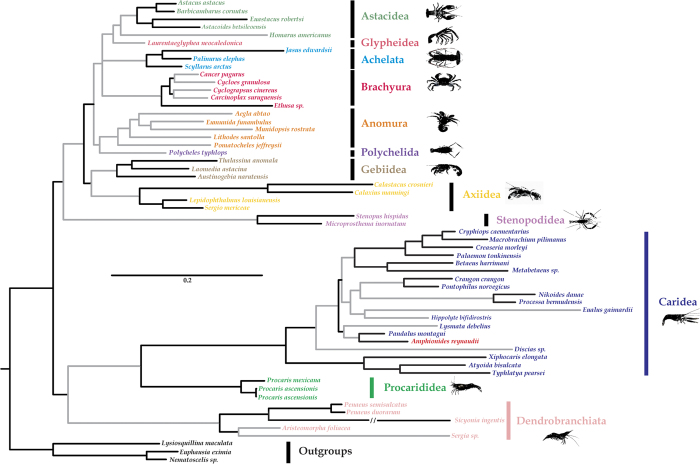
Bayesian phylogram for *Amphionides reynaudii* and selected decapods. Euphausiacea and Stomatopoda are used as outgroups, tree based on the concatenated dataset of 16 S rRNA, 18S rRNA, 28S rRNA and H3 genes. Nodes with Bayesian posterior probabilities >0.95 and maximum likelihood bootstrap values >75 are indicated as dark branches versus the other nodes with lower support values as grey branches.

**Figure 3:**
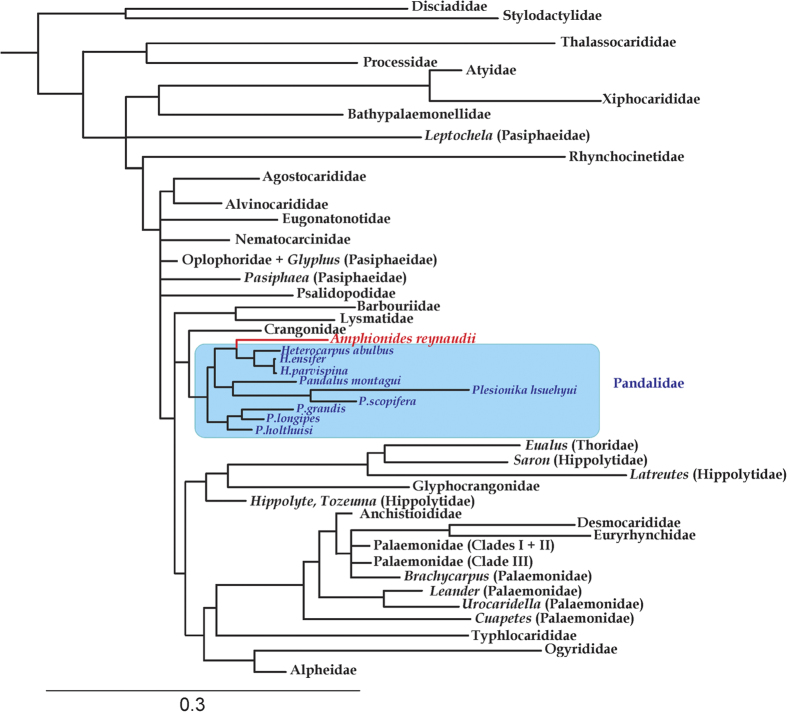
Simplified Bayesian phylogram for caridean shrimp taxa and *Amphionides reyaudii*. Branches are truncated at family level or genera for non-monophyletic families, palaemonid clades follows De Grave *et al.*^37^, full species level tree is presented in Supplementary Figure S1. Tree based on the concatenated dataset of 16 S rRNA and 18 S rRNA genes.

Of the six *Amphionides* larvae obtained, the largest specimen (CtsL 13.6 mm, NTOU M01872, Fig. 1) was used for DNA analysis (GenBank accession nos. KT699039-KT699042). Total genomic DNA was extracted from the abdominal muscles using QIAGEN^®^ DNeasy Blood and Tissue Kit (Cat. No. 69504, Valencia, CA) following the protocol of the manufacturer. Universal primer sets were used to amplify partial sequences of the targeted genes by polymerase chain reaction (PCR): 16 S rRNA (16Sar/16Sbr, ~500 bp^32^), 18 S rRNA (18SA/18SL, 18SC/18SY, 18SB/18SO, ~1900 bp^17^), 28 S rRNA (28 S Rd1.2a/28 S Rd4.2b, 28 S A/28 S B, 28 S Rd4.5a/28 S 6.2b, ~1800 bp^33^) and H3 (H3AF/H3AR, ~330 bp^34^). All amplifications were performed in 25 μL reactions with 50–250 ng of the DNA templates using TaKaRa *Tag*^TM^ kit, included 2.5 μL of 10 x polymerase buffer (Mg^2+^plus), 0.5 μL of 2.5 mM of deoxyribonucleotide mixture (dNTPs) and 0.5 U of Taq polymerase (5 U/μL) and additional 10–25 mM magnesium chloride (MgCl_2_) (depending on gene). Finally 0.5 μL of 10 μM for each primer (MISSION BIOTECH, Taipei, Taiwan) were added and supplemented with sterile double-distilled water (ddH_2_O) to a total volume of 25 μL volume. PCR cycling conditions were as follows: 5 min at 95 °C for first denaturation, then 40 cycles of 30 sec at 94 °C, 40 sec at 46–52 °C (depending on genes) and 40 sec at 72 °C, with final extension for 10 min at 72 °C. PCR products of correct size and quality checked by 1% agarose gel were sent to a commercial company (MISSION BIOTECH) for sequencing. The same PCR primer sets were also employed for sequencing on an ABI 3730 Genetic Analyzer (Applied Biosystems, Center for Integrated BioSystems, Logan, UT, USA). SeqMan Pro^TM^ (LASERGENE^®^; DNASTAR, Madison, WI, USA) was used to clean and edit the sequences for contig assembly.

### DNA analysis

The phylogenetic relationship of *Amphionides* was elucidated by incorporating the sequences into a published 16 S, 18 S, 28 S and H3 dataset^18^, to which the infraorder Glypheidea was added (*Laurentaeglyphea neocaledonica*, GenBank accession nos. HQ241517, HQ241528, HQ241562, KT699043). The latest overall classification scheme of Decapoda was followed^35^, with family level systematics within Caridea updated^23^,^29^,^36^,^37^.

To resolve the phylogenetic position of *Amphionides* in relation to Caridea, sequence data were analysed along with a comprehensive caridean 16 S and 18 S dataset^17^, but only including taxa with both markers. Additional caridean species with sequence data of both 16 S and 18 S sequences available from GenBank were also incorporated in the analysis. Included as outgroups in the analysis were representatives of all suborders and infraorders of Decapoda^35^, but are not shown on the tree in Supplementary Figure S1.

Sequences were aligned with MAFFT v.7^38^. GBlocks v.0.91b website server (http://molevol.cmima.csic.es/castresana/Gblocks_server.html)^39^ was used to remove divergent regions and poorly aligned positions in the DNA datasets. After elimination of highly divergent regions, each gene alignment for the four-gene dataset was trimmed to 406 bp of 16 S rRNA, 1675 bp of 18 S rRNA, 1477 bp of 28 S rRNA and 326 bp of H3. In the two-gene dataset, the 16 S and 18 S sequences were trimmed to 466 bp and 1768 bp, respectively. These alignments were then concatenated into the four-gene and two-gene datasets. The best model of DNA substitution and parameters for individual alignment (16 S and 18 S) of the two-gene dataset was determined by jModelTest v.2.1.3^40^ based on Akaike’s criterion (AIC). Two analytical methods were employed to construct the phylogenetic trees: maximum likelihood (ML) using RAxML v.7.2.6 (Randomized Axelerated Maximum Likelihood^41^) and Bayesian inference (BI) by MrBayes v. 3.2.1^42^. ML analysis settings followed the model of general time reversible with a gamma distribution and proportion invariant (GTRGAMMAI) for the two partitioned datasets. Branch confidence of the tree topology was assessed using 1000 bootstrap replicates (MLb)^43^. Three independent BI runs were performed with 10 million generations and sampled one tree every 1000 generations. Model parameters from jModelTest were applied on the partitioned dataset. Tracer v.1.6^44^ was used to evaluate the convergence of Bayesian runs. Observed likelihood (-LnL) scores was employed to determine the burn-ins and stable distributions for the two datasets. The majority rule trees from the four- and two-gene datasets were constructed from the remaining trees to estimate the posterior probabilities (Pp).

The approximately unbiased (AU) test^45^ was carried out as implemented in Consel v.0.1i^46^ to test for two hypotheses: (i) *Amphionides* does not belong to the Caridea, and (ii) *Amphionides* does not belong to Pandalidae. The same two concatenated datasets were run for the ML analysis based on GTRGAMMA model in RAxML v.7.2.6. The alternative tree topologies were also constructed and optimized by RAxML. The algorithm ‘-f g’ was used to compute per site log-likelihood scores for those hypothetical trees and evaluate the significance (*P* < 0.05) with the present phylogenetic trees in Consel.

## Additional Information

**How to cite this article**: De Grave, S. *et al.* Phylogenetics reveals the crustacean order Amphionidacea to be larval shrimps (Decapoda: Caridea). *Sci. Rep.* **5**, 17464; doi: 10.1038/srep17464 (2015).

## Supplementary Material

Supplementary Information

## References

[b1] Martin J. W. & Davis G. E. An updated classification of the recent Crustacea. Contr. Sc. 39, 1–124 (2001).

[b2] Richter S. & Scholtz G. Phylogenetic analysis of the Malacostraca (Crustacea). J. Zool. Syst. Evol. Research 39, 113–136 (2001).

[b3] Fransen C. H. J. M. Order Amphionidacea Williamson. 1973. Treatise on Zoology-Anatomy, taxonomy, biology. The Crustacea, vol 9A. Schram F. R. & von Vaupel Klein J. C. (eds.) (Brill, Leiden, 2010).

[b4] Heegaard P. Larvae of decapod Crustacea. The Amphionidae. Dana Rep. 77, 1–82 (1969).

[b5] Williamson D. I. *Amphionides reynaudii* (H. Milne Edwards), representative of a proposed new order of eucaridean Malacostraca. Crustaceana 25, 35–50 (1973).

[b6] Schram F. R. Crustacea. (Oxford University Press, Oxford, 1986).

[b7] Holthuis L. B. The recent genera of the caridean and stenopodidean shrimps (Crustacea, Decapoda) with an appendix on the order Amphionidacea. (Nationaal Natuurhistorisch Museum, Leiden, 1993).

[b8] Koeppel E. Beiträge zur kenntnis der gattung Amphion. Arch. Naturg. 68, 262–298 (1902).

[b9] Spence Bate C. Report on the Crustacea Macrura collected by the Challenger during the years 1873-76. Rep. Sc. Res. Voyage Challenger 24, 1–942 (1888).

[b10] Gurney R. Larvae of decapod Crustacea. 2. Amphionidae. Discovery Rep. 12, 392–399 (1936).

[b11] Gurney R. Larvae of decapod Crustacea. London: Ray Society. 306 pp.(1942).

[b12] Christoffersen M. L. Phylogenetic systematics of the Eucarida (Crustacea Malacostraca). Rev. Bras. Zool. 5, 325–351 (1988).

[b13] Legg D. A., Sutton M. D. & Edgecombe G. D. Arthropod fossil data increase congruence of morphological and molecular phylogenies. Nat. Commun. 4, 2485 (2013) 24077329 10.1038/ncomms3485

[b14] Vereshchaka A. L. New family and superfamily for a deep-sea caridean shrimp from the Galathea collections. J. Crust. Biol. 17, 361–373 (1997).

[b15] De Grave S., Chu K. H. & Chan T.-Y. On the systematic position of *Galatheacaris abyssalis* (Decapoda: Galatheacarididae). J. Crust. Biol. 30, 521–527 (2010).

[b16] Bracken-Grissom H. D., Felder D. L., Vollmer N. L., Martin J. W. & Crandall K. A. Phylogenetics links monster larva to deep-sea shrimp. Ecol. Evol. 2, 2367–2373 (2012). 23145324 10.1002/ece3.347 PMC3492765

[b17] Bracken H. D., De Grave S. & Felder D. L. Phylogeny of the infraorder Caridea based on mitchondrial and nuclear genes (Crustacea: Decapoda). Decapod Crustacean Phylogenetics. Martin J. W., Crandall K. A. & Felder D. L. (eds.) (CRC Press, Boca Raton, 2009).

[b18] Bracken H. D., De Grave S., Toon A., Felder D. L. & Crandall K. A. Phylogenetic position, systematic status, and divergence time of the Procarididea. Zool. Scr. 39, 198–212 (2010).

[b19] Li C. P., De Grave S., Lei H. C., Chan T.-Y. & Chu K. H. Molecular systematics of caridean shrimps based on five nuclear genes: Implications for superfamily classification. Zool. Anz. 250, 270–279 (2011).

[b20] Zimmer C. *Amphionides valdiviae* n.g, n. sp. Zool. Anz. 28, 225–228 (1904).

[b21] Bauer R. T. Remarkable shrimps. Adaptations and natural history of the Carideans. (University of Oklahoma Press, Norman, 2004).

[b22] Kutschera V., Maas A., Waloszek D., Haug C. & Haug J. T. Re-study of larval stages of *Amphionides reynaudii* (Malacostraca: Eucarida) with modern imaging techniques. J. Crust. Biol. 32, 916–930 (2012).

[b23] De Grave S. & Fransen C. H. J. M. Carideorum Catalogus: The recent species of the dendrobranchiate, stenopodidean, procarididean and caridean shrimps (Crustacea, Decapoda). Zool. Med. 85, 195–589 (2011).

[b24] Bartilotti C., Calado R., Rhyne A. & Dos Santos A. Shedding light on the larval genus *Eretmocaris*: morphological larval features of two closely related trans-isthmian *Lysmata* species (Decapoda: Caridea: Hippolytidae) described on the basis of laboratory cultured material. Helg. Mar. Res. 66, 97–115 (2012).

[b25] Landeira J. M., Chan T.-Y., Aguilar-Soto N., Jiang G. C. & Yang C. H. Description of the decapodid stage of *Plesionika narval* (Fabricius, 1787) (Decapoda: Caridea: Pandalidae) identified by DNA barcoding. J. Crust. Biol. 34, 377–387 (2014).

[b26] Lindley J. A. & Hernández F. The occurrence in waters around the Canary and Cape Verde Islands of *Amphionides reynaudii*, the sole species of the order Amphionidacea (Crustacea: Eucarida). Rev. Acad. Can. Cienc. 11, 113–119 (1999).

[b27] Fransen C. H. J. M. & Landeira J. M. New data on the mesopelagic shrimp community of the Canary Islands region. Crustaceana 85, 385–414 (2012).

[b28] Landeira J. M., Lozano-Soldevilla F. & Hernández-León S. Temporal and alongshore distribution of decapod larvae in the oceanic island of Gran Canaria (NW Africa). J. Plankton Res. 35, 309–322 (2013).

[b29] Ariza A. V., Garijo J. C., Landeira J. M., Bordes F. & Hernández-León S. Migrant biomass and respiratory carbon flux by zooplankton and micronekton in the north east Atlantic Ocean (Canary Islands). Progr. Oceanogr. 134, 330–342 (2015).

[b30] De Grave S. Li C. P., Tsang L. M., Chu K. H. & Chan T.-Y. Unweaving hippolytoid systematics (Crustacea, Decapoda, Hippolytidae): resurrection of several families. Zool. Scr. 43, 496–507 (2014).

[b31] d’Udekem d’Acoz C. Inventaire et distribution des crustacés décapodes de l’Atlantique nord-oriental, de la Méditerranée et des eaux continetales adjacentes au nord de 25°N. Patr. Nat. (MNHN/SPN) 40, 1–383 (1999).

[b32] Palumbi S. R. *et al.* A simple fool’s guide to PCR, v2.0. Spec. Publ. U. Hawaii Dep. Zool. Kewalo Mar. Lab. 1–23 (1991).

[b33] Whiting M. F. Mecoptera is paraphyletic: multiple genes and phylogeny of Mecoptera and Siphonaptera. Zool. Scr. 31, 93–104 (2002).

[b34] Colgan D. J. *et al.* Histone 3 and U2 snRNA DNA sequences and arthropod molecular evolution. Austr. J. Zool. 46, 419–437 (1998).

[b35] De Grave S. *et al.* A classification of living and fossil genera of decapod crustaceans. Raffles Bull. Zool. S21, 1–109 (2009).

[b36] Wong M. L., Pérez-Moreno J. L., Chan T.-Y., Frank T. M. & Bracken-Grissom H. D. Phylogenetic and transcriptomic analyses reveal the evolution of bioluminescence and light detection in marine deep-sea shrimps of the family Oplophoridae (Crustacea: Decapoda). Mol. Phylogenet. Evol. 83, 278–292 (2015). 25482362 10.1016/j.ympev.2014.11.013

[b37] De Grave S., Fransen C. H. J. M. & Page T. J. Let’s be pals again: major systematic changes in Palaemonidae (Crustacea: Decapoda). PeerJ 3, e1167 (2015). 26339545 10.7717/peerj.1167 PMC4558070

[b38] Katoh K. & Standley D. M. MAFFT multiple sequence alignment software version 7: improvements in performance and usability. Mol. Biol. Evol. 30, 772–780 (2013). 23329690 10.1093/molbev/mst010 PMC3603318

[b39] Castresana J. Selection of conserved blocks from multiple alignments for their use in phylogenetic analysis. Mol. Biol. Evol. 17, 540–552 (2000). 10742046 10.1093/oxfordjournals.molbev.a026334

[b40] Darriba D., Taboada G. L., Doallo R. & Posada D. jModelTest 2: more models, new heuristics and parallel computing. Nat. Methods 9, 772 (2012). 22847109 10.1038/nmeth.2109 PMC4594756

[b41] Stamatakis A. RAxML-VI-HPC: maximum likelihood based phylogenetic analyses with thousands of taxa and mixed models. Bioinformatics 22, 2688–2690 (2006). 16928733 10.1093/bioinformatics/btl446

[b42] Ronquist F. & Huelsenbeck J. P. MRBAYES 3: Bayesian phylogenetic inference under mixed models. Bioinformatics 19, 1572–1574 (2003). 12912839 10.1093/bioinformatics/btg180

[b43] Felsenstein J. Confidence limits in phylogenies: an approach using the bootstrap. Evol. 39, 783–791 (1985). 10.1111/j.1558-5646.1985.tb00420.x 28561359

[b44] Rambaut A., Suchard M. A., Xie D. & Drummond A. J. (2014) Tracer v1.6, Available from http://beast.bio.ed.ac.uk/Tracer (2014).

[b45] Shimodaira H. An approximately unbiased test of phylogenetic tree selection. Syst. Biol. 51, 492–508 (2002). 12079646 10.1080/10635150290069913

[b46] Shimodaira H. & Hasegawa M. CONSEL: for assessing the confidence of phylogenetic tree selection. Bioinformatics 17, 1246–1247 (2001). 11751242 10.1093/bioinformatics/17.12.1246

