# Supplementary material for: Phylogenetics reveals the crustacean order Amphionidacea to be larval shrimps (Decapoda: Caridea)
Source: Sci Rep. 2015 Dec 8;5:17464. doi: 10.1038/srep17464 (PMC4672333; doi:10.1038/srep17464)
Supplement: Supplementary Information [file srep17464-s1.pdf]

Phylogenetics reveals the crustacean order Amphionidacea to be larval shrimps (Decapoda: Caridea)

Sammy De Grave[1], Tin-Yam Chan[2,3], Ka Hou Chu[4,5], Chien-Hui Yang[2,]* & José M. Landeira[6]

[1] Oxford University Museum of Natural History, Oxford, United Kingdom

[2] Institute of Marine Biology, National Taiwan Ocean University, Keelung 20224, Taiwan

[3] Center of Excellence for the Oceans, National Taiwan Ocean University, Keelung 20224, Taiwan

[4] Simon F.S. Li Marine Science Laboratory, School of Life Sciences, The Chinese University of Hong Kong, Hong Kong, China

[5] Shenzhen Research Institute, The Chinese University Hong Kong, Shenzhen, China

[6] Graduate School of Fisheries Sciences, Hokkaido University, 3-1 Minato, Hakodate, Hokkaido 041-8611, Japan

Supplementary Figure S1 Full Bayesian phylogram for caridean shrimp taxa and *Amphionides reyaudii*. Tree based on the concatenated dataset of 16S rRNA and 18S rRNA genes. Bayesian posterior probabilities (Pp) and maximum likelihood bootstrap values (MLb) are represented as percentages above or below the branches. Values <50% are not shown. Representatives of all other infra/suborders within Decapoda were used as outgroups (see Supplementary Table S2), but are not shown.

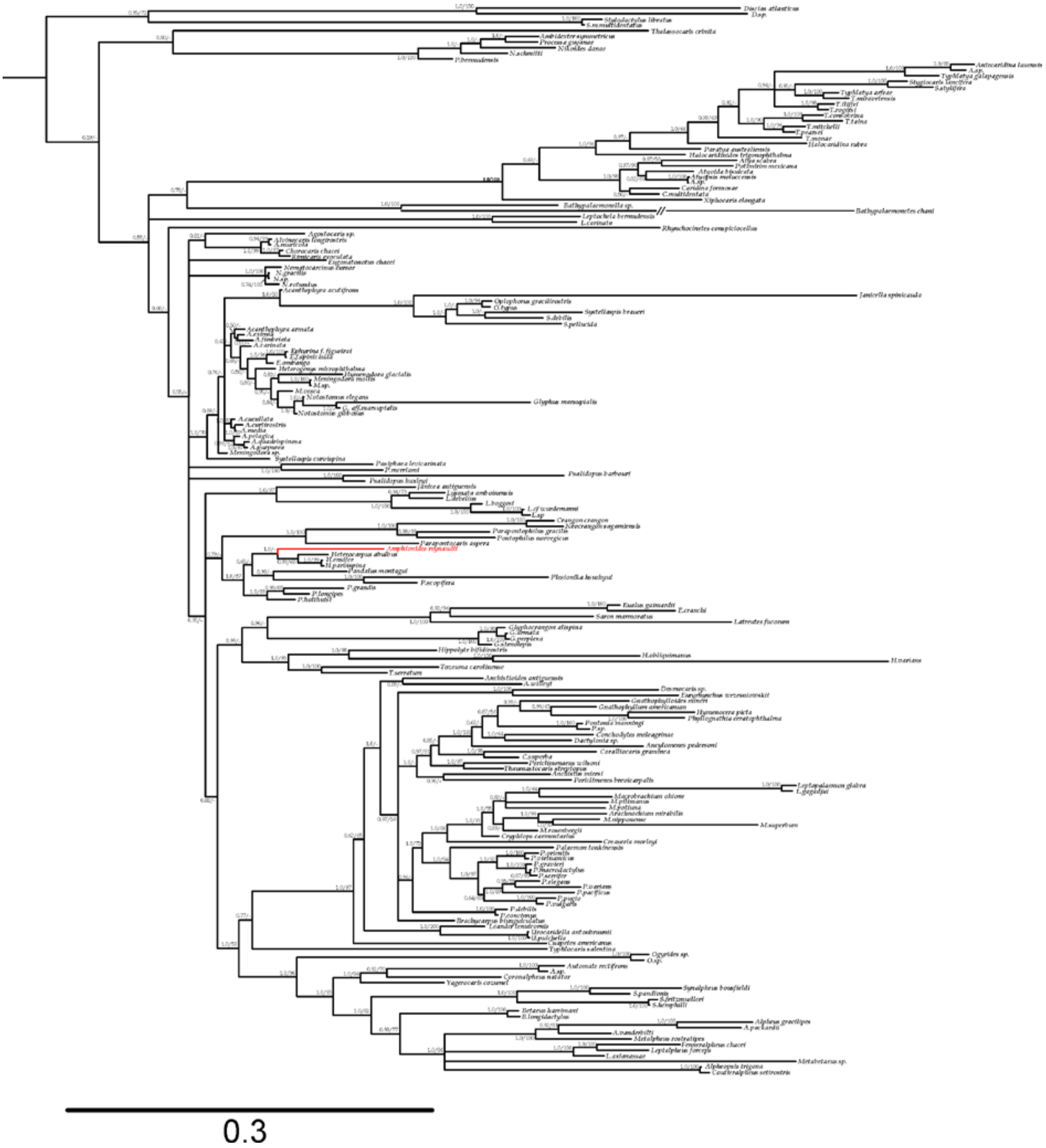

Supplementary Table S2 Taxa and GenBank accession numbers used in the two-marker analysis. The latest overall classification scheme of Decapoda was followed[35], with family level systematics within Caridea updated[23,29,36,37].

Taxon	GenBank Nos.	
	**16S**	**18S**
**Amphionidacea**		
Amphionides reynaudii	KT699039	KT699040
**Euphausiacea**		
Euphausia sp.	EU868655	EU868746
**Decapod -Dendrobranchiata**		
Penaeus semisulcatus	DQ079731	DQ079766
Sergia sp.	EU868710	EU868807
**Decapoda-Achelata**		
Palinurus argus	AF337966	AY743955
**Decapoda-Anomura**		
Pagurus longicarpus	AF150756	AF436018
**Decapoda-Astacidea**		
Enoplometopus occidentalis	AY583892	AY583966
Procambarus clarkii	DQ666844	AF436001
**Decapoda-Axiidea**		
Lepidophthalmus louisianensis	DQ079717	DQ079751
**Decapoda-Brachyura**		
Dromia dehaani	AY583899	AY583972
**Decapoda-Gebiidea**		
Upogebia affinis	AF436047	AF436007
**Decapoda-Glypheidea**		
Laurentaeglyphea neocaledonica	HQ241517	HQ241528
**Decapoda-Polychelida**		
Polycheles typhlops	EU920932	EU920950
**Decapoda-Procarididea**		
Procaris ascensionis	GQ487494	GQ487502
Procaris mexicana	EU868715	EU868811
**Decapoda-Stenopodidea**		
Stenopus hispidus	AY583884	AY743957
**Decapoda-Caridea**		
ALPHEIDAE		
Alpheopsis trigona	EU868633	EU868723
Alpheus gracilipes	DQ642885	DQ642859
Alpheus packardii	EU868630	EU868720
Alpheus vanderbilti	EU868639	EU868730
Automate rectifrons	EU868631	EU868721
Automate sp.	EU868635	EU868725
Betaeus harrimani	FJ943434	FJ943440
Betaeus longidactylus	JX010752	JF346263
Coronalpheus natator	EU868636	EU868727
Coutieralpheus sp.	EU868637	EU868728
Fenneralpheus chacei	EU868638	EU868729
Leptalpheus axianassae	EU868671	EU868764
Leptalpheus forceps	EU868670	EU868763
Metabetaeus sp.	FJ943435	FJ943441
Metalpheus rostratipes	KF023115	JF346264
Synalpheus bousfieldi	EU868646	EU868737
Synalpheus fritzmuelleri	EU868642	EU868733

Synalpheus hemphilli	EU868643	EU868734
Synalpheus pandionis	EU868647	EU868738
Yagerocaris cozumel	EU868645	EU868736
BARBOURIIDAE		
Janicea antiguensis	KF023112	JF346262
HIPPOLYTIDAE		
Hippolyte bifidirostris	EU920927	EU920939
Hippolyte obliquimanus	EU868661	EU868752
Hippolyte varians	EU868662	EU868753
Latreutes fucorum	EU868664	EU868755
Saron marmoratus	KF023102	JF346244
Tozeuma carolinense	EU868669	EU868760
Tozeuma serratum	EU868668	EU868759
THORIDAE		
Eualus gaimardii	EU920923	EU920940
Eualus cranchii	EU868667	EU868758
LYSMATIDAE		
Lysmata boggessi	DQ079719	DQ079753
Lysmata debelius	DQ079718	DQ079752
Lysmata cf. *wurdemanni*	EU868666	EU868757
Lysmata sp.	EU868665	EU868756
OGYRIDIDAE		
Ogyrides sp.1	EU868679	EU868772
Ogyrides sp.2	EU868680	EU868773
ATYIDAE		
Antecaridina lauensis	EU123851	HE801016
Antecaridina sp.	EF173754	EF173850
Atya scabra	EU868632	EU868722
Atyoida bisulcata	DQ079704	DQ079738
Atyopsis moluccensis	DQ681281	JF346252
Atyopsis sp.	EU868634	EU868724
Caridina formosae	DQ478496	GQ131924
Caridina multidentata	DQ478505	JF346236
Halocaridina rubra	EF173749	EF173848
Halocaridinides trigonophthalma	EF173752	EF173849
Paratya australiensis	EU868640	EU868731
Potimirim mexicana	EU868641	EU868732
Stygiocaris lancifera	EU123831	HE801019
Stygiocaris stylifera	EU123839	HE801020
Typhlatya arfeae	HE801000	HE801025
Typhlatya consobrina	HE801011	HE801028
Typhlatya iliffei	HE800998	HE801023
Typhlatya galapagensis	HE800991	HE801017
Typhlatya miravetensis	HE800999	HE801024
Typhlatya mitchelli	EU868644	EU868735
Typhlatya monae	HE801001	HE801026
Typhlatya pearsei	DQ079735	DQ079770
Typhlatya rogersi	HE801002	HE801027
Typhlatya taina	HE801005	HE801029
AGOSTOCARIDIDAE		
Agostocaris sp.	EU868626	EU868716
ALVINOCARIDIDAE		
Alvinocaris longirostris	JQ035659	JF346247
Alvinocaris muricola	EU868628	EU868718
Chorocaris chacei	AM087922	AM087653
Rimicaris exoculata	AM076958	AM087652
DISCIADIDAE		
Discias atlanticus	EU868652	EU868743

Discias sp.	EU920921	EU920941
BATHYPALAEMONELLIDAE		
Bathypalaemonella sp.	EU868648	EU868739
Bathypalaemonetes chani	GQ131910	GQ131933
CRANGONIDAE		
Crangon crangon	EU868649	EU868740
Neocrangon sagamiensis	KF023106	GQ131936
Parapontocaris aspera	KF023107	JF346235
Parapontophilus gracilis	EU868650	EU868741
Pontophilus norvegicus	GQ487496	GQ487504
GLYPHOCRANGONIDAE		
Glyphocrangon alispina	EU868656	EU868747
Glyphocrangon armata	HQ241513	HQ241525
Glyphocrangon perplexa	KF023104	JF346255
Glyphocrangon stenolepis	KF023103	JF346241
EUGONATONOTIDAE		
Eugonatonotus chacei	EU868653	EU868744
NEMATOCARCINIDAE		
Nematocarcinus cursor	EU868673	EU868766
Nematocarcinus gracilis	KP075927	KP075825
Nematocarcinus rotundus	EU868672	EU868765
Nematocarcinus sp.	GQ131895	GQ131919
RHYNCHOCINETIDAE		
Rhynchocinetes conspiciocellus	GQ131914	JF346243
XIPHOCARIDIDAE		
Xiphocaris elongata	EU868714	EU868809
OPLOPHORIDAE		
Acanthephyra acutifrons	KP075878	KP075829
Acanthephyra armata	KP075894	KP075786
Acanthephyra carinata	KP075896	KP075798
Acanthephyra cucullata	KP075893	KP075809
Acanthephyra curtirostris	EU868676	EU868769
Acanthephyra eximia	KP075897	KP075823
Acanthephyra fimbriata	KP075895	KP075788
Acanthephyra media	KP075892	KP075805
Acanthephyra pelagica	KP075881	KP075808
Acanthephyra purpurea	EU868677	EU868770
Acanthephyra quadrispinosa	KP075886	KP075821
Ephyrina figueirai	AM076960	AM087654
Ephyrina figueirai spinicauda	KP075911	KP075800
Ephyrina ombango	KP075915	KP075810
Heterogenys microphthalma	KP075898	KP075787
Hymenodora glacialis	KP075908	KP075828
Janicella spinicauda	KP075933	KP075858
Meningodora mollis	KP075910	KP075783
Meningodora vesca	KP075907	KP075791
Meningodora sp.1	EU868678	EU868771
Meningodora sp.2	GQ131890	GQ131931
Notostomus elegans	KP075901	KP075797
Notostomus gibbosus	KP075905	GQ131917
Oplophorus gracilirostris	KP075922	KP075848
Oplophorus typus	KP075923	KP075835
Systellaspis braueri	KP075926	KP075853
Systellaspis curvispina	KP075916	KP075784
Systellaspis debilis	EU868682	EU868775
Systellaspis pellucida	KP075925	KP075857
ANCHISTIOIDIDAE		
Anchistioides antiguensis	EU868629	EU868719

Anchistioides willeyi	DQ642883	DQ642857
DESMOCARIDIDAE		
Desmocaris sp.	EU868651	EU868742
EURYRHYNCHIDAE		
Euryrhynchus wrzesniowskii	EU868654	EU868745
PALAEMONIDAE		
Anchistus miersi	DQ642877	DQ642851
Ancylomenes pedersoni	KM071741	AY743954
Arachnochium mirabilis	KC515033	KC515052
Brachycarpus biunguiculatus	EU868686	EU868778
Conchodytes meleagrinae	KC515051	EF540837
Coralliocaris graminea	KF738361	AM083319
Coralliocaris superba	DQ642880	DQ642854
Creaseria morleyi	DQ079710	DQ079746
Cuapetes americanus	EU868701	EU868795
Cryphiops caementarius	DQ079711	DQ079747
Dactylonia sp.	DQ642876	DQ642850
Gnathophylloides mineri	EU868659	EU868750
Gnathophyllum americanum	EU868660	EU868751
Hymenocera picta	EU868663	EU868754
Leander tenuicornis	EU868690	EU868783
Leptopalaemon gagadjui	EU868693	EU868787
Leptopalaemon glabra	EF588318	EU249463
Macrobrachium nipponense	HQ830201	DQ531769
Macrobrachium ohione	EU868694	EU868788
Macrobrachium pilimanus	GQ487497	GQ487505
Macrobrachium potiuna	DQ079721	DQ079756
Macrobrachium rosenbergii	GQ131903	GQ131934
Macrobrachium superbum	KC515041	KC515055
Palaemon concinnus	KC515043	KC515056
Palaemon debilis	KC515044	KC515057
Palaemon elegans	EU868696	EU868790
Palaemon gravieri	KC515045	KC515058
Palaemon macrodactylus	DQ642875	DQ642849
Palaemon pacificus	KC515046	KC515059
Palaemon serrifer	KC515048	KC515060
Palaemon orientis	KC515036	KC515053
Palaemon pugio	EU868697	EU868791
Palaemon tonkinensis	EU920920	EU920937
Palaemon varians	JQ042301	KC763180
Palaemon vietnamicus	KC515037	KC515054
Palaemon vulgaris	JQ042300	AY743941
Periclimenaeus wilsoni	EU868702	EU868797
Periclimenes brevicarpalis	DQ642879	DQ642853
Phyllognathia ceratophthalma	DQ642873	DQ642847
Pontonia manningi	EU868705	EU868800
Pontonia sp.	EU868706	EU868801
Thaumastocaris streptopus	DQ642878	DQ642852
Urocaridella antonbruunii	KC515049	KC515061
Urocaridella pulchella	KC515050	KC515062
TYPHLOCARIDIDAE		
Typhlocaris salentina	EU868713	EU868808
PANDALIDAE		
Heterocarpus abulbus	KF023186	JF346257
Heterocarpus ensifer	KP075930	KP075776
Heterocarpus parvispina	HQ241514	HQ241526
Pandalus montagui	GQ487498	GQ487506
Plesionika grandis	KF023113	JF346258

Plesionika holthuisi	EU868703	EU868798
Plesionika hsuehyui	AM076968	AM083322
Plesionika longipes	EU868704	EU868799
Plesionika scopifera	HQ241519	HQ241530
THALASSOCARIDIDAE		
Thalassocaris crinita	EU868712	EU868810
PASIPHAEIDAE		
Glyphus marsupialis	KP075861	KP075775
Glyphus aff. *marsupialis*	KP075902	KP075772
Leptochela bermudensis	EU868691	EU868785
Leptochela carinata	EU868692	EU868786
Pasiphaea levicarinata	GQ131899	GQ131921
Pasiphaea merriami	EU868700	EU868796
PROCESSIDAE		
Ambidexter symmetricus	EU868683	EU868776
Nikoides danae	FJ943436	FJ943442
Nikoides schmitti	EU868695	EU868789
Processa bermudensis	GQ487499	GQ487507
Processa guyanae	EU868708	EU868803
PSALIDOPODIDAE		
Psalidopus barbouri	EU868709	EU868804
Psalidopus huxleyi	GQ131898	GQ131923
STYLODACTYLIDAE		
Stylodactylus libratus	AM076943	AM083323
Stylodactylus multidentatus multidentatus	EU868711	EU868806
